# Temozolomide induces senescence but not apoptosis in human melanoma cells

**DOI:** 10.1038/sj.bjc.6604017

**Published:** 2007-10-30

**Authors:** N M Mhaidat, X D Zhang, J Allen, K A Avery-Kiejda, R J Scott, P Hersey

**Affiliations:** 1 Immunology and Oncology Unit, Room 443, David Madison Building, Newcastle, New South Wales, Australia; 2 Discipline of Medical Genetics, School of Biomedical Sciences, Faculty of Health, University of Newcastle, New South Wales 2300, Australia; 3 Centenary Institute of Cancer Medicine and Biology, University of Sydney, Camperdown, New South Wales 2050, Australia

**Keywords:** temozolomide, p53, melanoma, senescence, apoptosis

## Abstract

Temozolomide (TMZ), a DNA alkylating agent used in the treatment of melanoma, is believed to mediate its effect by addition of a methyl group to the O^6^ position of guanine in DNA. Resistance to the agent may be in part due to the activity of O^6^-methylguanine-DNA methyl transferase (MGMT). In the present study, we show that sensitivity of melanoma cells to TMZ was dependent on their p53 status and levels of MGMT. Analysis of the mechanisms underlying reduced viability showed no evidence for induction of apoptosis even though marked levels of apoptosis was seen in TK6 lymphoma cells. Sensitivity of melanoma cells was associated with p53-dependent G2/M cell cycle arrest and induction of senescence. To verify the role of p53, the assays were repeated in presence of pifithrin-*α*, an inhibitor of p53. This resulted in increased viability of melanoma cells with wild-type p53 and reversed G2/M cell cycle arrest. Paradoxically, apoptosis was increased in melanoma but decreased as expected in TK6 lymphoma cells. These results are consistent with the view that TMZ is relatively ineffective against melanoma due to defective apoptotic signalling resulting from activation of p53. The nature of the defects in apoptotic signalling remains to be explored.

Melanoma continues to increase in incidence in many parts of the world and remains among the top six cancers as a cause of death and morbidity. However, there has been little progress in the medical treatment of metastatic melanoma because of the absence of effective systemic therapies. A variety of mechanisms that may account for resistance of melanoma cells to chemotherapy have been described. These include decreased drug uptake into the cells, increased drug efflux, intracellular drug inactivation, repair of drug-induced damage, or resistance to drug-induced apoptosis (Pepponi *et al*, 2003; Plummer *et al*, 2005).

Alkylating agents are among the most widely used chemotherapeutic agents for the treatment of metastatic melanoma. Temozolomide (TMZ), a second-generation imidazoletetrazinone derivative, is known to have clinical activity against melanoma (Pepponi *et al*, 2003; Plummer *et al*, 2005). By the oral route, it has a bioavailability of almost 100% and is rapidly absorbed. At physiological pH, TMZ hydrolyses to the cytotoxic methylating product, 3-methyl-(triazen-1-yl)imidazole-4-carboxamide (Middlemas *et al*, 2000; Plummer *et al*, 2005). The cytotoxicity of TMZ is believed to be mediated mainly through the addition of a methyl group to the O^6^ position of guanine (G) in genomic DNA (Tentori *et al*, 1997; Middlemas *et al*, 2000; Plummer *et al*, 2005). The methyl group in O^6^-methylguanine can be removed by O^6^-methylguanine-DNA methyl transferase (MGMT) (Tentori *et al*, 1997; Hirose *et al*, 2001). Unless repair occurs, O^6^-methylguanine is a cytotoxic lesion due to mispairing of G with thymine (T) during the next cycle of DNA replication (Middlemas *et al*, 2000). This triggers the DNA mismatch repair (MMR) system that removes, but then reinserts the T during repair synthesis (Hirose *et al*, 2001).

Futile cycles of MMR induced by GT mismatches have been reported to lead to a variety of outcomes, such as G2/M cell cycle arrest, cellular senescence, and apoptosis in TMZ-treated cells (Hirose *et al*, 2001; Gunther *et al*, 2003). p53 is thought to play a critical role in determining the sensitivity of cells to TMZ because the futile DNA repair cycles induces the accumulation of p53 protein, and transcriptional targets involved in cellular processes such as proliferation and apoptosis (Tentori *et al*, 1997; D’Atri *et al*, 1998; Hirose *et al*, 2001). For example, p53 has been shown to affect both the duration of cell cycle arrest and the fate of human glioblastoma cells treated with TMZ (Hirose *et al*, 2001). Mutations in the p53 gene are a common feature in many types of cancers, and are frequently associated with resistance to chemotherapeutic agents including TMZ (Tentori *et al*, 1998; Hussein *et al*, 2003). In haematopoietic neoplasms, p53 status has been shown to be directly correlated with sensitivity to TMZ-induced apoptosis (Tentori *et al*, 1998).

To address the responses of melanoma to TMZ and to determine the role of p53, we employed melanoma cell lines that express wild type or mutant p53. We report in the present study that treatment of melanoma cells with TMZ resulted in p53-dependent reduction in cell viability, which was due to induction of G2/M cell cycle arrest and cellular senescence. No significant apoptotic events could be observed after treatment of melanoma with TMZ even when MGMT was inhibited.

## MATERIALS AND METHODS

### Cell lines

Human melanoma cell lines Me4405, IgR3, Mel-FH, Mel-CV, MM200, and SK-mel-28 were cultured as described previously (Zhang *et al*, 2001). TK6 and MT1 lymphoblastic cells were kindly provided by Dr Josef Jiricny, University of Zurich.

### Antibodies and other reagents

TMZ, kindly provided by the Schering-Plough Research Institute (NJ, USA), was prepared freshly in dimethyl sulphoxide (DMSO) at a final concentration of 0.1%. (v/v). MGMT, O^6^-benzylguanine (BG), 3-(4,5-dimethylthiazol-2-yl)-2,5-diphenyltetrazolium bromide (MTT), X-gal, potassium ferricyanide, potassium ferrocyanide, and propidium iodide (PI) were purchased from Sigma-Aldrich (Castle Hill, NSW, Australia). The rabbit polyclonal Ab against caspase-3, the mouse MAbs against poly(ADP-Ribose) polymerase (PARP), and p21 were purchased from Pharmingen (Marrickville, NSW, Australia). The MAb against p53 (clone BP53-12) was purchased from Upstate Biotechnology (Lake Placid, NY, USA). The anti-phospho-AMPK-*α* (Thr172) was purchased from Cell Signaling Technology.

### Cell viability assays

These assays were performed using MTT as described previously (Wu *et al*, 2005).

### Apoptosis

Quantitation of apoptotic cells by measurement of sub-G1 DNA content using the PI method or by Annexin-V staining was carried out as described previously (Zhang *et al*, 2001; Gillespie *et al*, 2004).

### PI uptake assay

The PI uptake assay was performed as described previously (Zhang *et al*, 2006).

### Cell cycle analysis

Sub-confluent, exponentially proliferating cells were treated with TMZ and were harvested, counted and 1 × 10^6^ cells were stained with PI buffer (10*μ*g ml^−1^ PI, 1% tri-sodium citrate, 0.1% Triton X-100, 100 *μ*M NaCl). Cell cycle distribution was determined using a FACscan flow cytometer and Cell Quest analysis software.

### Mitochondrial membrane potential (ΔΨm)

Changes in the mitochondrial membrane potential were measured as described previously (Zhang *et al*, 2006).

### Colony formation assays

Melanoma cells were seeded in 6-well culture plates at fixed densities (500 cells per well) 16–24 h before the addition of TMZ. At the end of the assay (12–15 days after treatment), cells were fixed with methanol and stained with crystal violet. Colonies with 50 or more cells were counted using phase contrast microscopy.

### Western blot analysis

Western blots were performed as described previously (Zhang *et al*, 1999).

### Transfection of siRNA

The human melanoma cell line, IgR3, was seeded at a density of 2 × 10^5^ cells per well in a 6-well plate 24 h prior to transfection. Cells were transfected with 100 nM p21 siRNA or a non-specific target (control) siRNA (siGENOME ON-TARGET plus SMARTpool duplex, Dharmacon, Lafayette, CO, USA) using Lipofectamine 2000 (Invitrogen, Mount Waverley, VIC, Australia) according to the manufacturers’ instructions. After 24 h of transfection, cells were treated with 100 *μ*M of TMZ or the control (0.1% DMSO) for 72 h, then harvested for western blots or cell-cycle distribution analysis.

### SA-*β*-gal assay

The activity of *β*-galactosidase (*β*-gal) at a sub-optimal pH of 6.0 was measured as a biomarker of senescence as described elsewhere (Dimri *et al*, 1995).

### Statistical analysis

Data are expressed as mean±s.e. The statistical significance of intergroup differences in normally distributed continuous variables was determined using Student's *t*-test. *P*-values of less than 0.05 were considered statistically significant.

## RESULTS

### TMZ reduces cell viability and colony formation in melanoma cells

To examine the antitumour potential of TMZ, we performed cell viability analysis on a panel of melanoma cell lines exposed to TMZ. All cell lines apart from SK-mel-28 and Mel-FH express wild-type p53 as analysed by DNA sequencing (data not shown). Cells were treated with TMZ (0–500 *μ*M), either alone or in combination with BG at 10 *μ*M for 72 h and then cell growth was evaluated using MTT assay. As shown in Table 1, p53 status and the expression level of MGMT was associated with sensitivity to TMZ. MM200 and IgR3 cell lines (express wild-type p53 and low MGMT levels) showed comparable sensitivity to TMZ, with IC_50_ values of 23 and 22 *μ*M, while SK-mel-28 and Mel-FH (mutant-type p53 and high MGMT level) cell lines were resistant with IC_50_ values of >256 and >247 *μ*M, respectively. Mel-RM cell line, with wild-type p53 and high content of MGMT, was extremely resistant to TMZ. Pre-treatment with BG has significantly sensitised melanoma cells to TMZ-induced growth inhibition.

**Figure 1:**
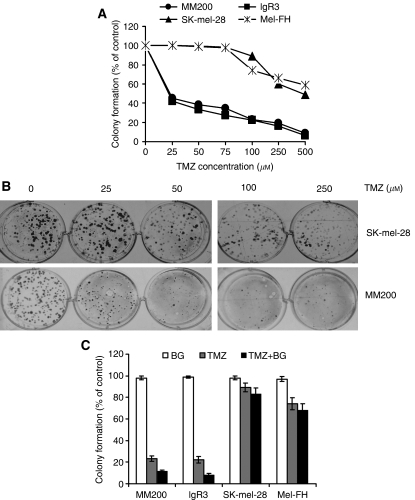
Colony formation in melanoma cell lines with wild type or mutant p53 at different concentration of temozolomide (TMZ). (**A** and **B**) MM200, IgR3, Mel-FH, and SK-mel-28 cells were seeded overnight before the treatment with or without TMZ at indicated doses for 12 days, and the numbers of colonies were quantitated. (**A**) Percentages of colony numbers relative to the negative controls. (**B**) Photographic images of the colonies. The data shown are representative of three experiments. (**C**) Cells were pre-treatment with or without benzylguanine (BG) at 10 *μ*M for 2 h followed by addition of TMZ at 100 *μ*M for 12 days. The data shown are mean±s.e. of three independent experiments.

Long-term effect of TMZ was studied in colony formation assays. Figure 1A and B show that TMZ inhibited colony formation in MM200 and IgR3 cell lines even when used at concentrations as low as 25 *μ*M. At 100 *μ*M, a clinically relevant concentration (Brada *et al*, 1999), colony formation was reduced to 22% of the control in IgR3 and 23% in MM200 cells. In contrast, inhibition of colony formation in SK-mel-28 and Mel-FH cells was observed only when TMZ was used at 75 *μ*M or higher. At clinically relevant dose of TMZ, the efficiency of colony formation was 89% of the control in SK-mel-28 and 74% in Mel-FH.

Similarly, cells with wild type or mutant p53 pre-treated with BG followed by treatment with TMZ for 12 days displayed lower numbers of colonies than those treated with TMZ alone. It is notable that even in the presence of BG, inhibition of colony formation by TMZ appeared to be more efficient in cells with wild-type p53 than those with mutated p53 (Figure 1C).

### No evidence for apoptosis in melanoma cells treated with TMZ

**Figure 2:**
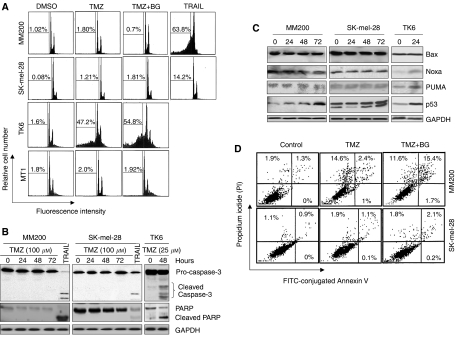
(**A**) Temozolomide (TMZ) does not induce apoptosis of melanoma cells. MM200 and SK-mel-28 cells were pre-treated with or without benzylguanine (BG) (10 *μ*M) for 2 h and then with TMZ at 100 *μ*M for 72 h. Apoptosis was assessed by measurement of sub-G1 DNA content. TRAIL (200 ng ml^−1^) was used as a positive control. MT1 and TK6 were seeded in medium containing TMZ (25 *μ*M) for 48 h before measurement of apoptosis. The data shown are representative of three individual experiments. (**B**) TMZ does not induce caspase-3 activation. MM200 and SK-mel-28 cells were treated with TMZ (100 *μ*M) for the indicated time periods. Processing of caspase-3 and its substrate PARP was measured by western blot analysis on whole cell lysates. TRAIL (200 ng ml^−1^) was used as a positive control. Western blot analysis of glyceraldehydes-3-phosphate dehydrogenase (GAPDH) level was included to show that equivalent amounts of protein were loaded in each lane. The data shown are representative of two individual experiments. (**C**) TMZ does not induce transcriptional activation of p53 target genes; Bax, Noxa, or PUMA. MM200 and SK-mel-28 cells were treated with TMZ (100 *μ*M) for the indicated time periods and the whole cell lysates were subjected to western blot analysis. The data shown are representative of two individual experiments. (**D**) TMZ-induced plasma membrane perturbation occurs earlier than externalisation of phosphatidylserine and augmented in presence of BG. MM200 and SK-mel-28 cells pre-treated with or without BG (10 *μ*M) followed by addition of TMZ (100 *μ*M) for 72 h were stained with fluorescein isothiocyanate (FITC)-conjugated Annexin-V and PI and analysed using flow cytometry. Numbers indicated the percentage of cells in each quadrant. The data shown are representative flow cytometry quadrant plot graphs of three individual experiments.

To study if the TMZ-induced reduction of cell viability of melanoma cells was due to induction of cell death, we studied cellular and molecular apoptotic events after treatment with TMZ for 72 h. As shown in Figure 2A and B, there was no significant apoptotic cell death after exposure to TMZ for 72 h as evidenced by lack of sub-G1 DNA fragmentation or activation of caspase-3 and processing of its substrate PARP. TRAIL is known to induce apoptosis of melanoma cells and was used as a positive control in these assays (16 h exposure to TRAIL) (Zhang *et al*, 2001). TK6 (sensitive) and MT1 (resistant) lymphoblastoid cells were used as a control for TMZ-induced apoptosis (D’Atri *et al*, 1998). Resistance to apoptosis in melanoma cells did not appear to be due to activation of intracellular survival signalling pathways mediated by Akt and ERK1/2 in that apoptosis was not detected when melanoma cells were pre-treated with inhibitors of these pathways (data not shown).

Most genotoxic agents including TMZ act primarily via p53 to induce apoptosis (Tentori *et al*, 1998; Bocangel *et al*, 2002), at least in part, by transcriptional activation of its target genes such as Bax, Noxa, and PUMA (Villunger *et al*, 2003). Figure 2C shows that Noxa and PUMA were constitutively expressed in the MM200 and SK-mel-28 melanoma cells and they and Bax were not upregulated after exposure to TMZ. p53 was weakly upregulated at 24 and 48 h in the MM200 cells but strongly upregulated in both cell lines by 72 h. These results contrast with studies on the TK6 lymphoblastoid cells in that TMZ-induced p53 and its downstream targets Noxa and PUMA by 24 h after treatment.

Uptake of PI into cells is a test for plasma membrane integrity and indicates necrosis when observed in the absence of evidence for apoptosis such as early plasma membrane externalisation of phosphatidylserine and detected by Annexin V staining (Walsh *et al*, 1998). Double staining with fluorescein isothiocyanate (FITC)-conjugated Annexin-V and PI revealed uptake of PI in 16% of MM200 cells and was not significant in SK-mel-28 cells (1.1%) after treatment with TMZ for 72 h (Figure 2D). Kinetic studies indicated that the majority of TMZ-treated MM200 cells did not become Annexin-V positive unless they first became PI positive (see Supplementary Figure S1).

Changes in mitochondrial membrane potential have been documented not only in apoptotic cell death, but also in other forms of cell death such as necrosis (Kroemer and Reed, 2000). Using a fluorescent cationic dye 5,5′,6,6′-tetrachloro-1,1′,3,3′-tetraethyl-benzamidazolocarbocyanin iodide, known as JC-1 (Nihal *et al*, 2005), we found that treatment with TMZ resulted in a reduction in ΔΨm in wild-type p53 (MM200) cells without being detected in mutant p53 (SK-mel-28) suggesting the absence of either apoptosis or necrosis of the latter (Supplementary Figure S2).

### TMZ induces G2/M cell cycle arrest in melanoma cells that is associated with accumulation of wild-type p53 and p21

**Figure 3:**
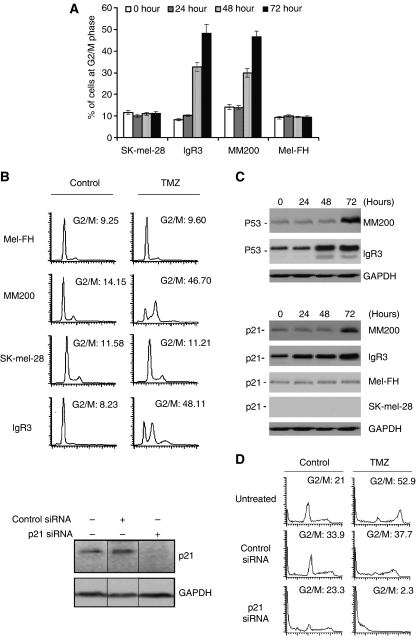
Temozolomide (TMZ) induces G2/M cell cycle arrest in association with accumulation of p53- and p21. (**A**) Melanoma cells treated with TMZ (100 *μ*M) for the indicated time periods were subjected to cell cycle analysis in flow cytometry. The data shown are percentages of the cells in G2/M phase (mean±s.e. of three individual experiments). (**B**) Representative flow cytometry histograms obtained from cells treated with TMZ (100 *μ*M) for 10 days. (**C**) TMZ upregulates p53 and p21. Whole cell lysates were subjected to western blot analysis. The data shown are representative of two individual experiments. (**D**) IgR3 cells were transfected with control siRNA or p21 siRNA and whole cell lysates were subjected to western blot analysis of p21 expression. Representative flow cytometry histograms of cell cycle distribution are shown for transfected cells which were treated with TMZ (100 *μ*M) for 72 h or treated with the control (DMSO). These results are representative of three independent experiments performed in duplicate.

We carried out cell cycle analysis of two cell lines with wild-type p53 (MM200 and IgR3) and two with mutant p53 (SK-mel-28 and Mel-FH) by DNA flow cytometric analysis. Results revealed that cell lines with wild-type p53 began to accumulate at the G2/M boundary 48 h after treatment with TMZ (Figure 3A). This G2/M arrest was sustained for at least 10 days after exposure to TMZ (Figure 3B) and was associated with the gradual appearance of hyperploid (>4 n DNA content) cells and the gradual decrease of cells with 2 n DNA content (G1 population). In contrast, little or no accumulation of cells at the G2/M boundary was observed in melanoma cell lines that expressed mutant p53.

Both p53 and p21 are known to play a key role in cell cycle regulation (D’Atri *et al*, 1998; Martin *et al*, 2005). As shown in Figure 3C, the levels of p53 were increased approximately 20-fold in MM200 and 8-fold in IgR3 cells 72 h after exposure to TMZ. Similarly, the levels of p21 were increased about 14-fold in MM200 and 6-fold in IgR3 at the same time point. In contrast, no significant change was found in the levels of p21 before and after treatment with TMZ in Mel-FH that expresses mutant p53. There was no detectable p21 expression in SK-mel-28 even in the presence of TMZ. Accumulation of p53 and p21 in MM200 and IgR3 cells persisted for at least 10 days after treatment (data not shown).

We investigated the role of p21 in induction of G2/M arrest by siRNA knockdown of p21 as described under Materials and Methods. As shown in Figure 3D, there was almost complete inhibition of G2/M arrest when p21 was knocked down by siRNA.

### Verification of the role of p53

**Figure 4:**
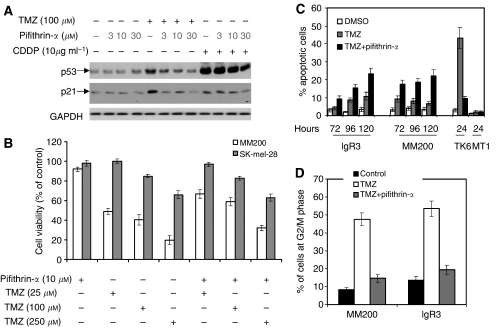
(**A**) Pifithrin-*α* induces dose-dependent inhibition of p53 transcriptional activity. MM200 cells were pre-treated with the indicated doses of pifithrin-*α* before the addition of temozolomide (TMZ) (100 *μ*M) for 72 h or CDDP (10 *μ*g ml^−1^) for 24 h as a positive control. Whole cell lysates were subjected to the western blot analysis for p53 and p21 expression. The data shown are representative of two individual experiments. (**B**) Pifithrin-*α* protects melanoma cells against TMZ-induced growth inhibition. MM200 and SK-mel-28 cells were pre-treated with or without pifithrin-*α* (10 *μ*M) for 2 h before the addition of TMZ at the indicated doses for another 72 h. Cell viability was assessed using MTT assay. The data shown represent percentages of viable cells relative to the negative controls (mean ± s.e. of three individual experiments). (**C**) Inhibition of p53 sensitises melanoma cells to TMZ-induced apoptosis. Melanoma and lymphoblastoma cells were treated with or without pifithrin-*α* for 3 h before adding TMZ for the indicated time points before measurement of apoptosis by the propidium iodide method. The data shown are representative of three individual experiments. (**D**) Pifithrin-*α* inhibits TMZ-induced G2/M cell cycle arrest. MM200 and IgR3 cells were pre-treated with pifithrin-*α* TMZ (10 *μ*M) for 3 h before adding TMZ (100 *μ*M) for 72 h. Cells were subjected to cell cycle analysis in flow cytometry. The data shown are percentages of the cells in G2/M phase (mean±s.e. of three individual experiments).

To verify the role of p53 in TMZ-induced anti-proliferative activity, we studied the effect of TMZ on cell proliferation and cell cycle distribution in presence and absence of the p53 inhibitor pifithrin-*α* [PFT-*α*, 2-(2-imino-4,5,6,7-tetrahydrobenzothiazol-3-yl)-1-(4-methylphenyl)ethanone] also called QB102. PFT-*α* has been reported to inhibit p53 function temporally *in vitro* (Komarov *et al*, 1999) by a reversible inhibition of p53-dependent transactivation of p53-responsive genes and to protect cells against a variety of genotoxic agents through inhibition of p53-mediated apoptosis (Culmsee *et al*, 2001). Figure 4A shows that after exposure to TMZ for 72 h, the expression of both p53 and p21 in MM200 cells was increased but were significantly inhibited when the PFT-*α* was used at 10 *μ*M (cisplatin at 10*ì*g ml^−1^ for the past 24 h was used as a positive control). p53 inhibitor partially protected MM200 against TMZ-induced growth inhibition when TMZ was used at 25 or 100 *μ*M (Figure 4B). The inhibitor however did not affect TMZ-induced growth inhibition of SK-mel-28 cells. PFT-*α* could not protect MM200 cells when TMZ was used at a higher dose (250 *μ*M) indicating that TMZ may induce p53-independent cell growth inhibition when used at high doses. As shown in Figure 4C, the levels of apoptosis induced by TMZ in MM200 and IgR3 cells were increased in the presence of PFT-*α*. In contrast, PFT-*α* inhibited apoptosis in the TK6 lymphoma cells. Moreover, Figure 4D shows that pre-treatment with PFT-*α* decreased the proportion of cells arrested at G2/M indicating that G2/M arrest was p53 dependent.

We asked whether cells treated with both TMZ and BG were inhibitable by PFT-*α* as BG might increase sensitivity to TMZ. MM200 cells were treated with BG at 10 *μ*M for 2 h and both TMZ (at 100 *μ*M) and PFT-*α* (at 10 *μ*M) added for 72 h. G2/M arrest was decreased from 48% in the absence of PFT-*α* to 16% in the presence of PFT, similar to the results shown in Figure 4D. Similarly, addition of BG did not increase or decrease the degree of apoptosis induced at 72 h in MM200 treated with TMZ plus PFT-*α* (data not shown).

### TMZ induces cellular senescence in melanoma cell lines with wild type or mutant p53

**Figure 5:**
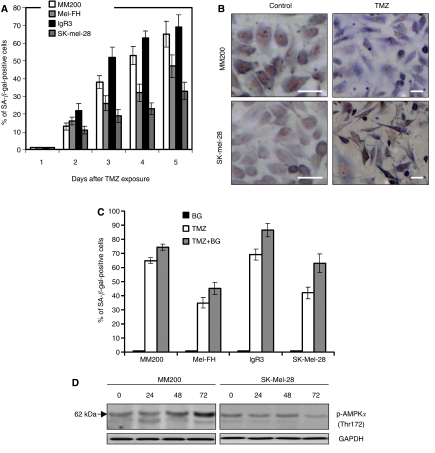
Benzylguanine (BG) enhanced temozolomide (TMZ)-induced cellular senescence. (**A**, **B** and **C**) Melanoma cells were seeded onto glass coverslips 16–24 h before the pre-treatment with or without BG (10 *μ*M) and then addition of TMZ (100 *μ*M) for the indicated time periods. Cells were stained with senescence-associated *β*-Gal stain solution, and were analysed using a microscope. The data shown are percentages of the *β*-Gal positive cells (mean±s.e. of three individual experiments) (**A** and **C**) and representative micrographs of three individual experiments (**B**). Bar=30 *μ*m. (**D**) Melanoma cells were treated with TMZ (100 *μ*M) for the indicated time points. Whole cell lysates were subjected to the western blots for phosphorylated AMP-activated protein kinase (AMPK) expression. Data shown are representative of two individual experiments.

Several cytotoxic agents including TMZ were reported to induce cellular senescence (Hirose *et al*, 2001; Schwarze *et al*, 2005). We examined if TMZ induces cellular senescence in melanoma cells treated with TMZ by measuring SA *β*-gal activity using immunohistochemistry. As shown in Figure 5A, SA *β*-gal positive cells could be detected as early as 48 h after treatment with TMZ in both p53-wild-type MM200 and IgR3, and p53-mutant SK-mel-28 and Mel-FH cells. At 5 days after exposure to TMZ, 65% of MM200 and 69% of IgR3 cells were positive, whereas only 35% of SK-Mel-28 and 42% of Mel-FH cells appeared to be positive for SA *β*-gal activity. Most of the SA *β*-gal positive cells also exhibited morphological characteristics of senescence with increased size and flattened shape (Figure 5B). The pre-treatment with BG increased the number of cells positive for SA *β*-gal irrespective of their p53 status (Figure 5C).

It was shown that activity of AMP-activated protein kinase (AMPK) is increased in cells undergoing senescence and its over-activation promoted senescence in primary human fibroblasts (Wang *et al*, 2003; Xiang *et al*, 2004). The level of phosphorylated AMPK was increased in MM200 cells but not SK-mel-28 after 48 h of treatment with TMZ and significantly accumulated after 72 h (Figure 5D).

## DISCUSSION

In the present study, sensitivity of melanoma cells to TMZ in assays of cell viability was shown to be dependent on the p53 status of the cells and their levels of MGMT. Melanoma cells with wild-type p53 had much reduced viability in the presence of TMZ compared to melanoma cells with mutated p53. Inhibition of MGMT with O^6^-benzylguanine resulted in further decrease in cell viability and this correlated with western blot analysis of MGMT levels in the melanoma cells.

Studies in animal models with alkylating agents have suggested that reduced viability would be the result of p53-mediated induction of proapoptotic BH3-only proteins leading to apoptosis via the mitochondrial pathway (Villunger *et al*, 2003). In the absence of apoptosis, cell death may result from necrosis, perhaps due to activation of PARP and consumption of ATP (Zong *et al*, 2004). This mechanism was shown to account for induction of necrosis by cisplatin in melanoma cells that were resistant to apoptosis (Zhang *et al*, 2006). In the present studies, there was no evidence that TMZ induced apoptosis in melanoma cells lines even though under the same experimental conditions, TMZ induced apoptosis in TK6 lymphoma cells at much lower concentration (25 *μ*M) than that used against the melanoma cells. Moreover, failure of apoptosis was not due to insensitivity of the cells to apoptosis as they were sensitive in varying degrees to TRAIL and cisplatin-induced apoptosis and in previous studies to docetaxel (Mhaidat *et al*, 2007).

Treatment with TMZ did not induce upregulation of Noxa or PUMA, which act as ‘sensor’ proteins in induction of apoptosis by DNA-damaging agents in a p53-dependent manner (Moll and Zaika, 2001). These proteins were however upregulated in the TK6 lymphoma cells. In addition, inhibition of survival pathways mediated by Akt and ERK1/2, which are known to protect melanoma from apoptosis induced by varying stimuli (Hagemann and Blank, 2001; Wu *et al*, 2005), did not potentiate TMZ-induced apoptosis. There was also relatively little evidence of cell necrosis (<10% of cells) based on PI uptake, lack of DNA fragmentation, delayed externalisation of phosphatidylserine, and absence of processing of PARP. These changes were mainly noted 48 h after treatment with TMZ consistent with the finding that the effect of TMZ does not take place until the second cell cycle O^6^-methyl G : T mismatches occur and MMR is triggered (D’Atri *et al*, 1998).

The main effect of TMZ on melanoma cells appeared to be p53-dependent cell cycle arrest in the G2/M phase. This was associated with upregulation of p21 in the cells with wild-type p53 but not in cells with mutated p53 where p21 levels were unchanged or undetectable after exposure to TMZ. These changes were associated with cellular senescence as shown by detection of SA-*β*-gal activity, morphological changes of senescent cells, and increased levels of AMP-activated protein kinase. Senescence appeared to be associated with p53 status in that the percentage of senescent cells was much higher in the cell lines with wild-type p53 than those with mutant p53 (for example, 65 and 35% in MM200 and SK-mel-28, respectively). The mechanism by which TMZ induces senescence in glioblastoma cells is thought to be by upregulation of p21 and inhibition of CDK2 (Hirose *et al*, 2001). In addition, p53-independent mechanisms may be involved but these are unclear. Busulfan was reported to induce senescence via the MAP kinase pathway (Probin *et al*, 2006). Similarly, in diterpine ester-treated melanoma cells, the senescence mechanism appeared to involve activation of the Ras–Raf–MEK–ERK pathway (Cozzi *et al*, 2006). Inhibition of MEK in the present study did not potentiate killing of the melanoma cells, which argues against this mechanism. It is unclear why p21 was upregulated whereas other p53 target genes such as PUMA and Noxa were not. Nevertheless, it is well known that there may be selective expression of p53 target genes due to cofactors such as the apoptosis stimulating proteins of p53 (Sullivan and Lu, 2007).

It is also unclear why inhibition of p53 with PFT-*α* resulted in an increase in apoptosis of human melanoma cells particularly as apoptosis of TK6 lymphoma cells was inhibited. Increased levels of apoptosis were also reported by others in glioma cells treated with PFT-*α*. It was speculated that this may be due to failure of the cells to undergo DNA repair during cell cycle arrest (Hirose *et al*, 2001; Xu *et al*, 2005). A similar interpretation could be placed on the current results. The actual mechanism of apoptosis is unknown except that it is presumably independent of p53-mediated pathways. We have reported elsewhere (Zhang *et al*, 2006) that melanoma may express smaller molecular weight isotypes of p53, and it is possible that these may act as a dominant negative regulator of some but not all p53 target genes. Studies on this aspect are in progress but if proven, it suggests that the resistance of melanoma to TMZ may in large part be due to abnormalities in p53-mediated regulation of its target genes.

In summary, the present studies confirm that the levels of MGMT play a role in resistance of melanoma to TMZ but also indicate that apoptotic cell death pathways are not activated by TMZ. Instead, reduced cell viability appeared to result from G2/M arrest and induction of senescence. Necrosis played a minor role in the effects of TMZ on melanoma. Resistance to apoptosis appears at least in part due to a p53-dependent mechanism perhaps resulting from cell cycle arrest and repair of DNA. These results provide new insights into the mechanism of action of TMZ and new approaches in its use against melanoma perhaps with agents which reactivate functions of p53 (Bossi and Sacchi, 2007).

## Figures and Tables

**Table 1:** Effect of TMZ or TMZ plus BG on the proliferation of melanoma cell lines

		**IC_50_ (*μ*M)^c^**	
**Cell line^a^**	**Relative expression of MGMT^b^ (fold)**	**TMZ**	**TMZ plus BG**
ME4405	2.11	36±1.9	29±2.3
SK-mel-28	16.78	>256	194±6.3
MM200	1.13	23±2.1	17±1.2
IgR3	0.99	22±2.3	16±0.9
Mel-CV	4.01	29±4.1	19±1.67
Mel-FH	14.88	>247	187±11.2
Mel-RM	12.32	>256	89±9.6

Abbreviations: TMZ=temozolomide; BG=benzylguanine; MGMT=O^6^-methylguanine-DNA methyl transferase; MTT=3-(4,5-dimethylthiazol-2-yl)-2,5-diphenyltetrazolium bromide; DMSO=dimethyl sulphoxide.

a Cells were incubated with graded concentration of TMZ for 72 h and then analysed for cell growth using MTT assay. BG at 10 *μ*m was added to the cell cultures 2 h before TMZ and maintained for the entire period of the assay. Control groups were either untreated or treated with BG or DMSO alone.

b The intensity of the MGMT bands was quantitated with the Bio-Rad VersaDoc image system (Bio-Rad, Regents Park, NSW, Australia). The relative expression of MGMT was determined by dividing the densitometric value of MGMT by that of the *α*-tubulin control.

c Drug concentration required to inhibit cell growth by 50%. Each value represents the mean±s.e. of three independent experiments performed with quadruplicate culture.
