# Supplementary figures and images for: Temozolomide induces senescence but not apoptosis in human melanoma cells
Source: Br J Cancer. 2007 Oct 30;97(9):1225–33. doi: 10.1038/sj.bjc.6604017 (PMC2360470; doi:10.1038/sj.bjc.6604017)

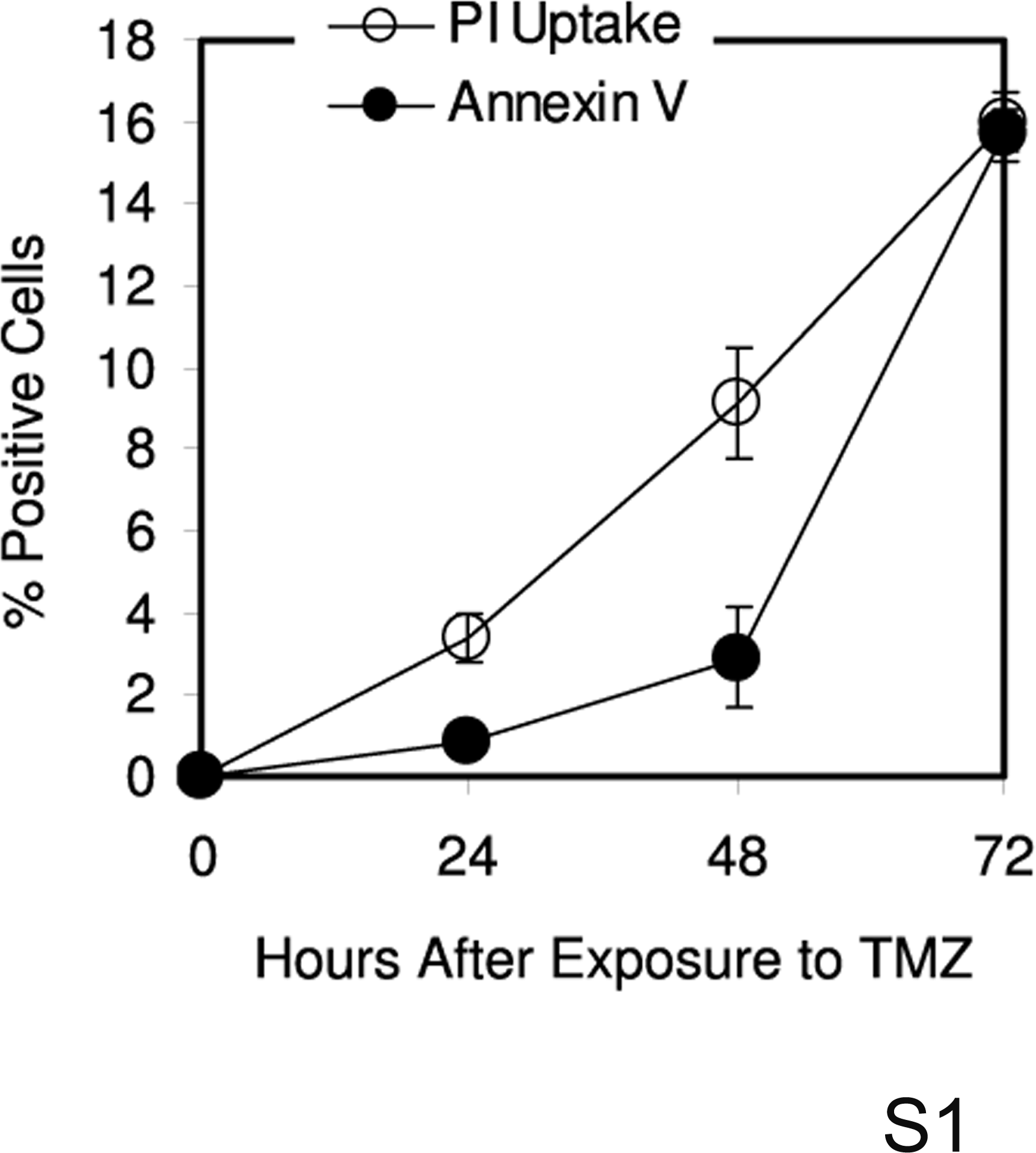

Supplement: Supplementary Figure S1 [file 6604017x1.tif]

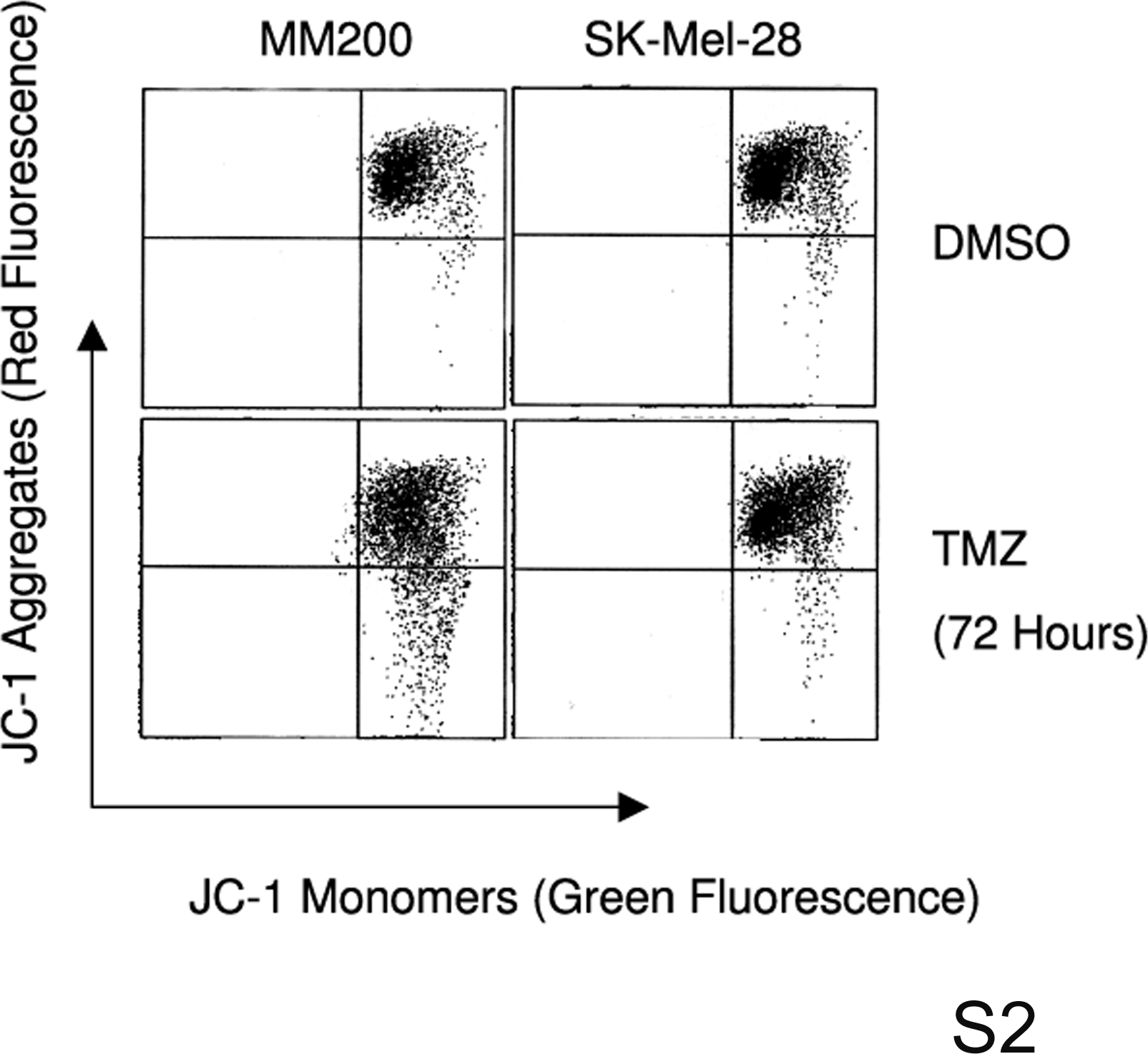

Supplement: Supplementary Figure S2 [file 6604017x2.tif]
