# Supplementary material for: Temozolomide induces senescence but not apoptosis in human melanoma cells
Source: Br J Cancer. 2007 Oct 30;97(9):1225–33. doi: 10.1038/sj.bjc.6604017 (PMC2360470; doi:10.1038/sj.bjc.6604017)
Supplement: Supplementary Figure Legends [file 6604017x3.doc]

**LEGENDS TO SUPPLEMENTARY FIGURES:**

S1 TMZ induces necrotic cell death at early treatment time. MM200 cells treated with TMZ (100μM) for the indicated time points were stained with FITC-conjugated Annexin-V and PI and analysed using flow cytometry. Data show the quantitation of Annexin-V or PI positive MM200 cells (mean ± SE of three individual experiments).

S2 TMZ induces damage to mitochondria. MM200 and SK-mel-28 cells were treated with TMZ at 100μM for indicated time periods followed by measurement of ΔΨm using JC-1 in flow cytometry. The data shown are representative of three individual experiments.
